# Germline *CDH1* mutations are a significant contributor to the high frequency of early-onset diffuse gastric cancer cases in New Zealand Māori

**DOI:** 10.1007/s10689-018-0080-8

**Published:** 2018-03-27

**Authors:** Christopher Hakkaart, Lis Ellison-Loschmann, Robert Day, Andrew Sporle, Jonathan Koea, Pauline Harawira, Soo Cheng, Michelle Gray, Tracey Whaanga, Neil Pearce, Parry Guilford

**Affiliations:** 1 Cancer Genetics Laboratory, Centre for Translational Cancer Research, University of Otago, P. O. Box 56, Dunedin, 9054 New Zealand; 2 Centre for Public Health Research, Massey University, Wellington, New Zealand; 3 Department of Statistics, The University of Auckland, Auckland, New Zealand; 4 Waitemata District Health Board, Auckland, New Zealand; 5 Kimihauora Health Centre, Tauranga, New Zealand; 6 Department of Medical Statistics, London School of Hygiene & Tropical Medicine, London, UK

**Keywords:** E-cadherin, CDH1, New Zealand, Māori, Hereditary diffuse gastric cancer, Genetic predisposition testing

## Abstract

New Zealand Māori have a considerably higher incidence of gastric cancer compared to non-Māori, and are one of the few populations worldwide with a higher prevalence of diffuse-type disease. Pathogenic germline *CDH1* mutations are causative of hereditary diffuse gastric cancer, a cancer predisposition syndrome primarily characterised by an extreme lifetime risk of developing diffuse gastric cancer. Pathogenic *CDH1* mutations are well described in Māori families in New Zealand. However, the contribution of these mutations to the high incidence of gastric cancer is unknown. We have used next-generation sequencing, Sanger sequencing, and Multiplex Ligation-dependent Probe Amplification to examine germline *CDH1* in an unselected series of 94 Māori gastric cancer patients and 200 healthy matched controls. Overall, 18% of all cases, 34% of cases diagnosed with diffuse-type gastric cancer, and 67% of cases diagnosed aged less than 45 years carried pathogenic *CDH1* mutations. After adjusting for the effect of screening known HDGC families, we estimate that 6% of all advanced gastric cancers and 13% of all advanced diffuse-type gastric cancers would carry germline *CDH1* mutations. Our results demonstrate that germline *CDH1* mutations are a significant contributor to the high frequency of diffuse gastric cancer in New Zealand Māori.

**Electronic supplementary material:**

The online version of this article (10.1007/s10689-018-0080-8) contains supplementary material, which is available to authorized users.

## Introduction

Although the worldwide incidence of gastric cancer has declined steadily over the past 4–5 decades, it remains the 5th most common cancer type worldwide [1]. The vast majority of gastric cancers are adenocarcinomas, which can be further subdivided into intestinal and diffuse type according to the Lauren classification [2]. Intestinal-type gastric cancer is more common in older patients and is more strongly associated with exposure to environmental risk factors, whereas diffuse-type gastric cancer is more associated with an earlier onset and a family history of the disease [2]. Typically, intestinal-type tumours predominate high-incidence geographic areas and account for much of the variation in gastric cancer incidence between groups [3].

As a whole, New Zealand is a country with a low incidence of gastric cancer [1]. However, Māori, the indigenous people of New Zealand (who comprise almost 15% of the total 4.5 million population) [4], experience disproportionate rates of gastric cancer compared to non-Māori [5]. The most recent data from the New Zealand Cancer Registry (NZCR) show Māori registration rates for gastric cancer are more than three times that of non-Māori (15.8 vs. 4.8 per 100,000, respectively) [6]. Additionally, on average, Māori develop gastric cancer 10 years younger than non-Māori and are one of the few populations worldwide with a higher incidence of diffuse-type disease [7, 8]. The reasons for these differences are largely unexplained.

Germline mutations in the gene *CDH1*, encoding the cell adhesion protein E-cadherin, are causative of the autosomal dominant cancer predisposition syndrome Hereditary Diffuse Gastric Cancer (HDGC) [9, 10]. Mutation carriers are predisposed to an extreme risk of developing diffuse-type gastric cancer from a relatively young age [11]. Based on HDGC families from around the world, the current estimated cumulative risk of developing diffuse gastric cancer by the age of 80 years is 70% for males (95% CI 59–80%) and 56% for females (95% CI 44–69%) [11]. In addition, women carrying *CDH1* mutations have a 42% (95% CI 23–68%) cumulative risk of developing lobular breast cancer by the age of 80 years [11]. In Family A, the large Māori kindred in which the first pathogenic *CDH1* mutation was identified, the overall penetrance of diffuse gastric cancer is approximately 70% [9]. In Western populations, it is estimated that 1% of all gastric cancers are caused by germline *CDH1* mutations [12].

Germline *CDH1* mutations have been well documented in Māori families in New Zealand [8, 9]. However, the contribution *CDH1* mutations make to the high incidence of diffuse gastric cancer in Māori is unknown. This paper presents the findings on the prevalence of cases with pathogenic *CDH1* mutations from a case-control study of gastric cancer conducted in the Māori population.

## Materials and methods

### Study participants

Study participants were from a New Zealand Māori population-based case-control study examining factors associated with gastric cancer risk [13]. Briefly, all Māori gastric cancers reported to the NZCR between 1 February 2009 and 31 October 2013 were followed up, with a sample of whole blood obtained from consenting participants who were well enough. The control group were a population-based random sample of individuals aged over 18 years who self-identified as Māori on the New Zealand electoral roll. Sequenced controls were matched to cases by gender and 5 year age bands. The study was granted ethics approval by the New Zealand Multi-region Ethics Committee (ref: MEC/08/08/102/AM03). Informed written consent was obtained from all study participants. Full details describing the identification of study participants and the collection of samples are described in Ellison-Loschmann et al. [13].

### Sequencing library preparation

Duel-indexed amplicon sequencing libraries for germline *CDH1* were generated using a novel two-step PCR strategy. Briefly, in the first PCR step, the coding exons of *CDH1*, including their intron–exon borders and the proximal promoter, were amplified. *CDH1* primers were designed with an additional 18 bp of known non-specific sequence that was used as a priming site for the second reaction (Supplementary Table 1). PCR products from the same study participant were pooled in equal volumes and purified using AMPure XP beads. In the second PCR step, pooled PCR products were amplified using a unique pair of indexed primers designed to add sequences necessary for multiplex sequencing on an Illumina MiSeq (Supplementary Table 2). Full details describing the PCR reactions are available upon request.

Sample specific libraries were pooled and sequenced in batches across multiple MiSeq runs. To prepare the sequencing libraries, sample libraries were pooled in equal volumes, purified using AMPure XP beads, and quantified with the Qubit dsDNA HS Assay Kit. Sequencing libraries were run on a DNA7500 Bioanlayzer chip to determine the average library size. Libraries were sequenced on an Illumina MiSeq using either V2-500 cycle or V3-600 cycle reagent kits.

### Sequence analysis and annotation

Raw paired end reads were cleaned with Trimmomatic v.0.35 [14]. Cleaned reads were aligned to the human reference genome (GRCh37/hg19) using the Burrows–Wheeler Aligner v.0.7.10 [15]. Amplicons were sequenced to a minimum depth of 40 reads. Any amplicon that did not reach this threshold was sequenced again in a subsequent MiSeq run or Sanger sequenced. Single nucleotide variants and insertion/deletion variants were called using ‘The Genome Analysis Toolkit’ (GATK) v.3.6 [16]. The effects of variants were predicted using SnpEff v.4.2 [17]. Variants were annotated with minor allele frequencies from the Exome Aggregation Consortium (ExAC) [18], the 1000 Genomes Project [19], and the University of Washington’s Exome Sequencing Project (ESP6500) [20] databases. The functional consequences of missense variants were predicted in silico using SIFT [21], Provean [22], and PolyPhen2 [23].

### Classification on pathogenic variants

Nonsynonymous and noncoding variants with a minor allele frequency (MAF) of < 0.05 for cases or controls were considered rare. All rare variants were queried in ClinVar and published literature, and were classified according to the American College of Medical Genetics and Genomics guidelines [24].

### Variant validation

All rare variants were visually inspected using the Integrative Genomics Viewer [25]. Rare nonsynonymous and noncoding variants were confirmed by re-extracting DNA from blood samples and Sanger sequencing.

### Copy number analysis

Cases without pathogenic *CDH1* mutations were subsequently tested for copy number changes using Multiplex Ligation Dependent Probe Amplification (MLPA). The SALSA MLPA *CDH1* probe-mix (v.C1) was used according to the manufacturer’s instructions. Results were analysed using Coffalyser.Net software.

### Statistical analysis

Statistical tests were performed using R v.3.3.3 [26]. The significance of correlation between clinical characteristics and mutation status were tested using Fisher’s exact test.

## Results

### Characteristics of study participants

**Fig. 1:**
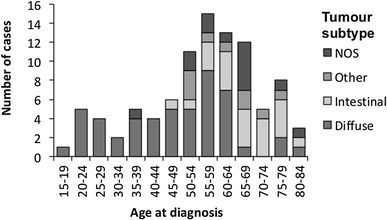
Tumour subtypes by age at diagnosis of gastric cancer in cases. *NOS* not otherwise specified

Germline *CDH1* was sequenced for 94 Māori gastric cancer patients and 200 healthy matched controls. The cases comprised 50 (53%) males and 44 (47%) females. The average age of cases at the time of gastric cancer diagnosis was 55.5 years (range 17–81 years). Tumour histology was available for 81/94 (86.2%) of cases. Out of these 81 cases, 50 (62%) were diffuse type, 22 (27%) were intestinal type, and 9 (11%) were other types. Of the 21 cases diagnosed in individuals younger than 45 years, 20 (95%) were diffuse type and one (5%) was unspecified (Fig. 1). The earliest intestinal-type tumour was diagnosed in a patient aged 49 years. The full clinical characteristics of the cases are presented in Table 1.

**Table 1:** Clinical characteristics of the study cases

	Total	
	n	%
**Total**	94	
**Gender**		
Male	50	53
Female	44	47
**Age at diagnosis (years)**		
< 45	21	22
45–59	32	34
60–74	30	32
> 75	11	12
**Tumour subtype**		
Diffuse	50	53
Intestinal	22	23
Other	9	10
NOS	13	14
**Tumour grade**		
Well differentiated	4	4
Moderately differentiated	10	11
Poorly differentiated	47	50
NOS	33	35
**Tumour site**		
Proximal	30	32
Distal	20	21
Mixed	4	4
Oesophageal junction	5	5
NOS	35	37
**Extent**		
Local	26	28
Lymph node involvement	20	21
Regional spread	7	7
Metastatic spread	13	14
NOS	28	30

*NOS*, not otherwise specified

Pathology reports from cases were reviewed for information that indicated prior genetic screening. Pathology reports from 15 cases described prophylactic gastrectomies, endoscopic screening, or noted *CDH1* mutation status as a part of the clinical pathway. As these procedures are offered to *CDH1* mutation carriers, it is likely these cases were known mutation carriers who had elected prophylactic surgery or who had foci of gastric cancer identified during endoscopic screening.

The controls comprised 104 (52%) males and 96 (48%) females. The average age of the controls was 57.6 years (range 19–84 years).

### Variants of uncertain significance

Four healthy controls were found to carry one of three variants of uncertain significance (c.88C>A (p.Pro30Thr), c.1214A>G (p.Asn405Thr), and c.2556G>T (p.Glu852Asp); Supplementary Table 3). To our knowledge, this is the first time these variants have been reported in the Māori population. No variants of uncertain significance were identified in the gastric cancer cases.

*CDH1* c.88C>A was identified in one healthy control aged 56 years. c.88C>A has been reported in population databases (MAF ≤ 0.001) and is most commonly classified as ‘likely benign’ in ClinVar. In silico prediction tools have conflicting interpretations of *CDH1* c.88C>A pathogenicity. Notably, germline *CDH1* c.88C>A has been reported in lobular breast and diffuse gastric cancer patients [27, 28], as well as two unrelated individuals with cleft lip with or without cleft palate, a developmental birth defect that is known to be overrepresented in *CDH1* mutation carriers [29, 30]. Additional evidence from in vitro assays indicate that the p.Pro30Thr mutation affects E-cadherin protein function and its subcellular localisation [30].

*CDH1* c.1214A>G (p.Asn405Ser) was identified in one healthy control (age 74 years) and c.2556G>T (p.Glu852Asp) was identified in two healthy controls (age 62 and 68 years). Both c.1214A>G and c.2556G>T are very rare in population variant databases (MAF < 0.0001) and are not described in published literature. In silico predictions do not support a pathogenic classification for either of these mutations.

### Pathogenic mutations

After reviewing all available information regarding the variants identified in this study, we classified five variants as pathogenic mutations (three nonsense, one frameshift, and one missense; Supplementary Table 4). The three nonsense mutations (c.190C>T (p.Gln64*), c.1792C>T (p.Arg598*), and c.2287G>T (p.Glu763*)) and one frameshift mutation (c.2381_2386insC (p.Arg796fs)) were identified in four cases each, while the deleterious missense mutation (c.2195G>A (p.Arg763Gln)) was identified in a single case (Table 2). The nonsense and frameshift mutations are known HDGC mutations that had previously been reported in Māori families in New Zealand [31]. c.2195G>A is a putative HDGC mutation that is located at the intracellular border of the cytoplasmic domain of E-cadherin and has been shown to create a new acceptor splice site and a large deletion in the E-cadherin protein [32]. c.2195G>A had previously been shown to be causative of HDGC in two families of northern European origin [33]. To our knowledge, this is the first time c.2195G>A has been reported in New Zealand Māori. No pathogenic mutations were identified in the controls.

**Table 2:** Clinical characteristics of Māori gastric cancer patients with pathogenic germline *CDH1* mutations

Identifier	Age^a^	Gender	Nucleotid change^b^	Protein change^b^	Subtype	Extent	Grade	Site
Y240	24	Female	c.190C>T	p.Gln64*	Diffuse	Local	NOS	NOS
Y382	29	Male	c.190C>T	p.Gln64*	Diffuse	Local	NOS	Proximal
Y704	48	Female	c.190C>T	p.Gln64*	Diffuse	Local	NOS	NOS
Y647	61	Female	c.190C>T	p.Gln64*	Diffuse	Local	NOS	Distal
Y649	20	Female	c.1792C>T	p.Arg598*	Diffuse	Local	NOS	NOS
Y579	23	Female	c.1792C>T	p.Arg598*	Diffuse	Local	NOS	NOS
Y709	24	Male	c.1792C>T	p.Arg598*	Diffuse	Local	Poorly differentiated	Proximal
Y255	29	Female	c.1792C>T	p.Arg598*	Diffuse	Local	NOS	Proximal
Y616	38	Female	c.2195G>A	p.Arg732Gln	Diffuse	Metastatic spread	Poorly differentiated	NOS
Y435	31	Female	c.2287G>T	p.Glu763*	Diffuse	Local	Poorly differentiated	Proximal
Y670	39	Female	c.2287G>T	p.Glu763*	Diffuse	Metastatic spread	NOS	NOS
Y706	41	Male	c.2287G>T	p.Glu763*	Diffuse	Local	NOS	NOS
Y335	50	Male	c.2287G>T	p.Glu763*	Diffuse	Local	NOS	NOS
Y638	17	Male	c.2381_2386insC	p.Arg796fs	Diffuse	Local	NOS	NOS
Y425	20	Male	c.2381_2386insC	p.Arg796fs	Diffuse	Local	NOS	NOS
Y666	26	Female	c.2381_2386insC	p.Arg796fs	Diffuse	Local	NOS	NOS
Y386	44	Male	c.2381_2386insC	p.Arg796fs	Diffuse	Local	NOS	NOS

*NOS* not otherwise specified

^a^Age at time of diagnosis

^b^Variant positions are reported in reference to NCBI RefSeq NM_004360.3 (mRNA) and NP_004351.1 (protein)

### Frequency of pathogenic mutations

**Fig. 2:**
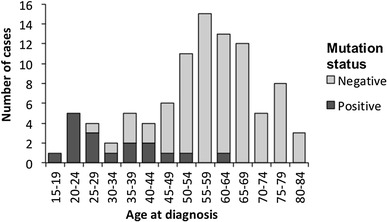
Frequency of pathogenic germline *CDH1* mutations by age at diagnosis of gastric cancer in cases

Overall, pathogenic germline *CDH1* mutations were identified in 17/94 (18%) of the total gastric cancer cases and 17/50 (34%) of diffuse gastric cancer cases (Table 3). The proportion of cases aged less than 45 years at diagnosis with a pathogenic germline *CDH1* mutation was 14/21 (67%). Only 3/73 (4%) of cases with mutations were aged 45 years and over (Fig. 2). The average age of diagnosis for mutation carriers was 33.2 years (range 17–61), and 60 years (range 28–81) for non-carriers. Of the pathogenic mutation carriers, 15/17 (88%) were diagnosed with early stage localised tumours, presumably subsequent to HDGC family mutation screening. The remaining two cases with pathogenic mutations were diagnosed with late stage metastatic disease and did not appear to be diagnosed as a result of being a known *CDH1* mutation carrier.

**Table 3:** Clinical characteristics of the study cases by mutation status

	*CDH1* mutation positive		*CDH1* mutation negative		p value
	n	%	n	%	
**Total**	17	18	77	82	
**Gender**					
Male	7	14	43	86	
Female	10	23	34	77	0.296
**Age at diagnosis (years)**					
< 45	14	67	7	33	
45–59	2	6	30	94	
60–74	1	3	29	97	
> 75	0	0	11	100	< 0.001
**Tumour subtype**					
Diffuse	17	34	33	66	
Intestinal	0	0	22	100	
Other	0	0	9	100	
NOS	0	0	13	100	< 0.001
**Tumour grade**					
Well differentiated	0	0	4	100	
Moderately differentiated	0	0	10	100	
Poorly differentiated	3	6	44	94	
NOS	14	42	19	58	< 0.001
**Tumour site**					
Proximal	4	13	26	87	
Distal	1	5	19	95	
Mixed	0	0	4	100	
Oesophageal junction	0	0	5	100	
NOS	12	34	23	66	0.048
**Extent**					
Local	15	58	11	42	
Lymph node involvement	0	0	20	100	
Regional spread	0	0	7	100	
Metastatic spread	2	15	11	85	
NOS	0	0	28	100	< 0.001

*NOS* not otherwise specified

Our data demonstrates that between 2009 and 2013, 18 and 34% of Māori gastric cancer and diffuse gastric cancer cases, respectively, carried pathogenic germline *CDH1* mutations. However, since the majority of these cases were diagnosed as a result of prior familial HDGC screening, these figures do not accurately represent the prevalence of pathogenic *CDH1* mutations in the Māori gastric cancer population. Without the targeted interventions offered to the 15 cases that were likely diagnosed as a result of being a known *CDH1* mutation carrier, it is unlikely they would have presented until their cancers had progressed and symptoms emerged. Accordingly, without prior genetic screening, the majority of *CDH1* mutation carriers identified during this study would have likely presented with advanced disease at a later date. Accounting for the number of Māori *CDH1* mutation carriers identified between 1998 and 2008 (P. Guilford, personal communication) and using a lifetime penetrance estimate of 70%, we estimate that in the absence of familial HDGC screening, germline *CDH1* mutations would account for 6% of all advanced Māori gastric cancers and 13% of all diffuse-type gastric cancers.

## Discussion

To our knowledge, this is the first study that has examined the frequency of gastric cancers that are attributable to germline *CDH1* mutations in a specific ethnic group. In keeping with previous studies, we observed a high proportion of diffuse gastric cancers, many of which were diagnosed in patients less than 45 years of age. Overall, 18% of all cases, 34% of cases diagnosed with diffuse-type gastric cancer, and 67% of cases diagnosed aged less than 45 years carried pathogenic *CDH1* mutations. Additionally, we estimate that in absence of screening, 6% of all advanced gastric cancer and 13% of all advanced diffuse-type gastric cancers in the Māori population would carry germline *CDH1* mutations.

Whether any of the variants of uncertain significance identified in healthy controls in this study impact on E-cadherin function and, consequently, have a role in gastric cancer predisposition remains to be clarified. In particular, the c.88C>A (p.Pro30Thr) mutation will require further evaluation to determine if it is associated with a hereditary cancer risk. Given the relative frequency of c.88C>A in the healthy population it is unlikely to be associated with an extreme risk of disease. However, as c.88C>A has been identified in unrelated cleft lip with or without cleft palate cases, and has been shown to cause defects in E-cadherin protein function and its subcellular localisation in vitro, it is plausible that the c.88C>A mutation has a deleterious effect on E-cadherin function in vivo and may represent a mutation with a low to moderate risk of disease.

It is unclear why the prevalence of pathogenic germline *CDH1* mutations is so high in the Māori gastric cancer population. Founder mutations have been identified as a common cause of cancer in some populations. Of note is the Ashkenazi Jewish population, for which approximately 2% of the general population carry one of three founder mutations in the tumour suppressor genes *BRCA1* and *BRCA2* [34]. Mutations in these genes are associated with an increased risk of both breast and ovarian cancer [35]. Subsequently, approximately 12% of breast cancers [36] and 40% [37] of ovarian cancers in the Ashkenazi Jewish population are attributable to these specific founder mutations. Similarly, a founder mutation in germline *CDH1* has previously been identified in multiple families from Newfoundland, Canada [32]. Interestingly, Newfoundland has an elevated rate of gastric cancer compared to the Canadian average and the regions these families come from are the highest-risk areas within the province [32, 38]. As yet, the exact contribution of *CDH1* mutations to the high rates of gastric cancer in the Newfoundland province is still to be determined.

Similar to the common mutation seen in Newfoundland, *CDH1* mutations could have arisen as founder mutations prior to the Māori migration to New Zealand. However, the relatively high number of distinct *CDH1* mutations (5 mutations in this study alone) suggests that, rather than being an illustration of a simple genetic bottleneck, *CDH1* mutations may have provided a selective advantage to mutation carriers in ancestral Māori populations.

One possible explanation is that *CDH1* mutation carriers may have a degree of innate resistance to infection with *Listeria monocytogenes*, a food-born pathogen that can cause gastroenteritis, meningitis, and miscarriage in pregnant women [39, 40]. *L. monocytogenes* is normally internalised into epithelial cells by a process requiring the binding of the bacterial protein internalin-A (*InlA*) to the N-terminus of the E-cadherin protein [40]. Some truncating E-cadherin mutations produce short soluble N-terminal peptides containing the *InlA* binding site that have been shown to act as decoy receptors for invading *L. monocytogenes* in vitro [39]. Alternatively, a reduction in functional E-cadherin available in mutation carriers may cause changes to the organisation of the cortical actin cytoskeleton which, in turn, may impact on the efficiency of endocytosis and the internalisation of *L. monocytogenes* or other pathogens [41].

The main purpose of our study was to determine the prevalence of pathogenic *CDH1* mutations in the Māori gastric cancer population. After reviewing the pathology reports from gastric cancer cases, the importance and impact of genetic screening for Māori became especially apparent. Notably, all 15 gastric cancer cases that were diagnosed as a result of interventions available to known *CDH1* mutation carriers were diagnosed with early-stage disease and were still alive five years post diagnosis (data not shown). In contrast, the two *CDH1* mutation carriers who did not appear to be known carriers were both diagnosed with late-stage metastatic disease and both died shortly after diagnosis. Clearly, clinical genetic screening and targeted interventions for *CDH1* mutation carriers is enabling timely and effective identification and management of mutation carriers in known HDGC families. Our findings suggest that, ideally, clinical germline *CDH1* testing should be incorporated into standard care for all Māori who present with early-onset diffuse gastric cancer.

As the most comprehensive study of germline *CDH1* mutations in a specific ethnic group, our study demonstrates the significant impact pathogenic *CDH1* mutations have on the high frequency of early-onset diffuse gastric cancer cases in the New Zealand Māori population. We highlight the importance of clinical genetic screening of HDGC families and the potential benefits of genetically screening all Māori who present with early-onset diffuse gastric cancer.

## Electronic supplementary material

Below is the link to the electronic supplementary material.

Supplementary material 1 (DOCX 36 KB)
