# Supplementary material for: Germline CDH1 mutations are a significant contributor to the high frequency of early-onset diffuse gastric cancer cases in New Zealand Māori
Source: Fam Cancer. 2018 Mar 27;18(1):83–90. doi: 10.1007/s10689-018-0080-8 (PMC6323075; doi:10.1007/s10689-018-0080-8)
Supplement: Supplementary file 1 — Supplementary material 1 (DOCX 36 KB) [file 10689_2018_80_MOESM1_ESM.docx]

**Supplementary Table 1** Oligonucleotides used for amplification of *CDH1* exons.

| **Primer Name** | **Oligonucleotide Sequence**^a^ |
| --- | --- |
| CDH1_Promoter_Fwd | **ACGACGCTCTTCCGATCTT**CGAACCCAGTGGAATCAGAAC |
| CDH1_Promoter_Rev | **CGTGTGCTCTTCCGATCT**ACAGGTGCTTTGCAGTTCCG |
| CDH1_Exon1_ Fwd | **ACGACGCTCTTCCGATCT**GAACTGCAAAGCACCTGTGA |
| CDH1_Exon1_Rev | **CGTGTGCTCTTCCGATCT**GTGACGACGGGAGAGGAAG |
| CDH1_Exon2_ Fwd | **ACGACGCTCTTCCGATCT**TTTCGGTGAGCAGGAGGGAA |
| CDH1_Exon2_Rev | **CGTGTGCTCTTCCGATCT**GGTGTGGGAGTGCAATTTCT |
| CDH1_Exon3_ Fwd | **ACGACGCTCTTCCGATCT**CGCTCTTTGGAGAAGGAATG |
| CDH1_Exon3_Rev | **CGTGTGCTCTTCCGATCT**CGGTACCAAGGCTGAGAAAC |
| CDH1_Exon4_ Fwd | **ACGACGCTCTTCCGATCT**TGATTGGTCATTTTGGTGGA |
| CDH1_Exon4_Rev | **CGTGTGCTCTTCCGATCT**GAATTAGTAAAGAAGGATCCCAAC |
| CDH1_Exon5_ Fwd | **ACGACGCTCTTCCGATCT**AGTGTTGGGATCCTTCTT |
| CDH1_Exon5_Rev | **CGTGTGCTCTTCCGATCT**CCCATCACTTCTCCTTAGCA |
| CDH1_Exon6_ Fwd | **ACGACGCTCTTCCGATCT**CAGCAGCACATGTGTGAGAAAAGTC |
| CDH1_Exon6_Rev | **CGTGTGCTCTTCCGATCT**GGAAGGATCAGCTTTAGTTACAC |
| CDH1_Exon7_ Fwd | **ACGACGCTCTTCCGATCT**CCAGTCCCAAAGTGCAGCTTGTCT |
| CDH1_Exon7_Rev | **CGTGTGCTCTTCCGATCT**CACCCTCTGGATCCTCCTGA |
| CDH1_Exon8_ Fwd | **ACGACGCTCTTCCGATCT**GTTCCGTGCCTAGAAGACA |
| CDH1_Exon8_Rev | **CGTGTGCTCTTCCGATCT**GCCATCTCAAGATGCTTGCT |
| CDH1_Exon9_ Fwd | **ACGACGCTCTTCCGATCT**TGACACATCTCTTTGCTCTGC |
| CDH1_Exon9_Rev | **CGTGTGCTCTTCCGATCT**AGAAGATACCAGGGGACAAGG |
| CDH1_Exon10_ Fwd | **ACGACGCTCTTCCGATCT**AACCACAGTTACTTTTGCACC |
| CDH1_Exon10_Rev | **CGTGTGCTCTTCCGATCT**AACCAGTTGCTGCAAGTCAG |
| CDH1_Exon11_ Fwd | **ACGACGCTCTTCCGATCT**TTCTAAAAGCCAGAGCTTGTCC |
| CDH1_Exon11_Rev | **CGTGTGCTCTTCCGATCT**GAGGGGCAAGGAACTGAACT |
| CDH1_Exon12_ Fwd | **ACGACGCTCTTCCGATCT**ACCACTGAAGAGCCAGGAC |
| CDH1_Exon12_Rev | **CGTGTGCTCTTCCGATCT**GAAATTGAAAGGTGGGGATCT |
| CDH1_Exon13_ Fwd | **ACGACGCTCTTCCGATCT**CGGGTGTCTTTAGTTCACTAGC |
| CDH1_Exon13_Rev | **CGTGTGCTCTTCCGATCT**TGGGAGTCTCTTTCCCACAT |
| CDH1_Exon14_ Fwd | **ACGACGCTCTTCCGATCT**GTGATAGCTGCTGCTTCTGG |
| CDH1_Exon14_Rev | **CGTGTGCTCTTCCGATCT**TGTTTCAAATGCCTACCTC |
| CDH1_Exon15_ Fwd | **ACGACGCTCTTCCGATCT**ACATAGCCCTGTGTGTATGAC |
| CDH1_Exon15_Rev | **CGTGTGCTCTTCCGATCT**AGAGATGAGCCATGCTTTGG |
| CDH1_Exon16_ Fwd | **ACGACGCTCTTCCGATCT**GATGACAGGTGTGCCCTTC |
| CDH1_Exon16_Rev | **CGTGTGCTCTTCCGATCT**CAGCAACGTGATTTCTGCAT |

^a^Oligonucleotide sequences shown in 5' to 3' direction. Bold characters denote the non-specific sequence that acts as a binding site for the second PCR reaction. Red characters denote the sequence targeting the *CDH1* gene.

**Supplementary Table 2** Oligonucleotides used for amplification of PCR products.

| **Primer Name** | **Oligonucleotide Sequence**^a^ |
| --- | --- |
| Adapter_Fwd | AATGATACGGCGACCACCGAGATCTACACXXXXXXACACTCTTTCCCTAC**ACGACGCTCTTCCGATCT** |
| Adapter_Rev | CAAGCAGAAGACGGCATACGAGATXXXXXXGTGACTGGAGTTCAGA**CGTGTGCTCTTCCGATC** |

^a^Oligonucleotide sequences shown in 5' to 3' direction. Underlined X's denote six base-pair index positions. Bold denotes bases that overlap step one PCR products.

**Supplementary Table 3** Variants of uncertain significance identified in germline *CDH1* in Māori gastric cancer cases and controls.

| **Exon** | **Nucleotide change^a^** | **Protein change^a^** | **SNP ID** | **Class** | **Cases (n=94)** | **Controls (n=200)** | **MAF ExAc^b^** | **MAF 1000G^c^** | **MAF ESP6500^d^** | **SIFT** | **PolyPhen2** | **Provean** |
| --- | --- | --- | --- | --- | --- | --- | --- | --- | --- | --- | --- | --- |
| 2 | c.88C>A | p.Pro30Thr | rs139866691 | Missense | 0 | 1 | 0.00094 | 0.0004 | 0.0010 | Tolerated | Probably damaging | Deleterious |
| 9 | c.1214A>G | p.Asn405Ser | . | Missense | 0 | 1 | 0.00003 | NA | NA | Tolerated | Possibly damaging | Neutral |
| 16 | c.2556G>T | p.Glu852Asp | . | Missense | 0 | 2 | 0.00002 | NA | NA | Tolerated | Benign | Neutral |

^a^Variant positions are reported in reference to NCBI RefSeq NM_004360.3 (mRNA) and NP_004351.1 (protein).

^b^Minor allele frequency (MAF) reported in the ExAc.

^c^MAF reported in the 1000 Genomes cohort (1000G).

^d^MAF reported in the Exome Variant Server, NHLBI GO Exome Sequencing Project (ESP6500).

NA, Not Available.

**Supplementary Table 4** Pathogenic germline *CDH1* mutations identified in Māori gastric cancer cases and controls.

| **Exon** | **Nucleotide change^a^** | **Protein change^a^** | **SNP ID** | **Class** | **Cases (n=94)** | **Controls (n=200)** | **MAF ExAc^b^** | **MAF 1000G^c^** | **MAF ESP6500^d^** |
| --- | --- | --- | --- | --- | --- | --- | --- | --- | --- |
| 3 | c.190C>T | p.Gln64* | . | Nonsense | 4 | 0 | NA | NA | NA |
| 12 | c.1792C>T | p.Arg598* | rs121964877 | Nonsense | 4 | 0 | NA | NA | NA |
| 14 | c.2195G>A | p.Arg732Gln | . | Missense | 1 | 0 | NA | NA | NA |
| 14 | c.2287G>T | p.Glu763* | . | Nonsense | 4 | 0 | NA | NA | NA |
| 15 | c.2381_2386insC | p.Arg796fs | . | Frameshift | 4 | 0 | NA | NA | NA |

^a^Variant positions are reported in reference to NCBI RefSeq NM_004360.3 (mRNA) and NP_004351.1 (protein).

^b^Minor allele frequency (MAF) reported in the ExAc.

^c^MAF reported in the 1000 Genomes cohort (1000G).

^d^MAF reported in the Exome Variant Server, NHLBI GO Exome Sequencing Project (ESP6500).

NA, Not available
